# Supplementary figures and images for: Calcineurin phosphatase activity regulates Varicella-Zoster Virus induced cell-cell fusion
Source: PLoS Pathog. 2020 Nov 20;16(11):e1009022. doi: 10.1371/journal.ppat.1009022 (PMC7717522; doi:10.1371/journal.ppat.1009022)

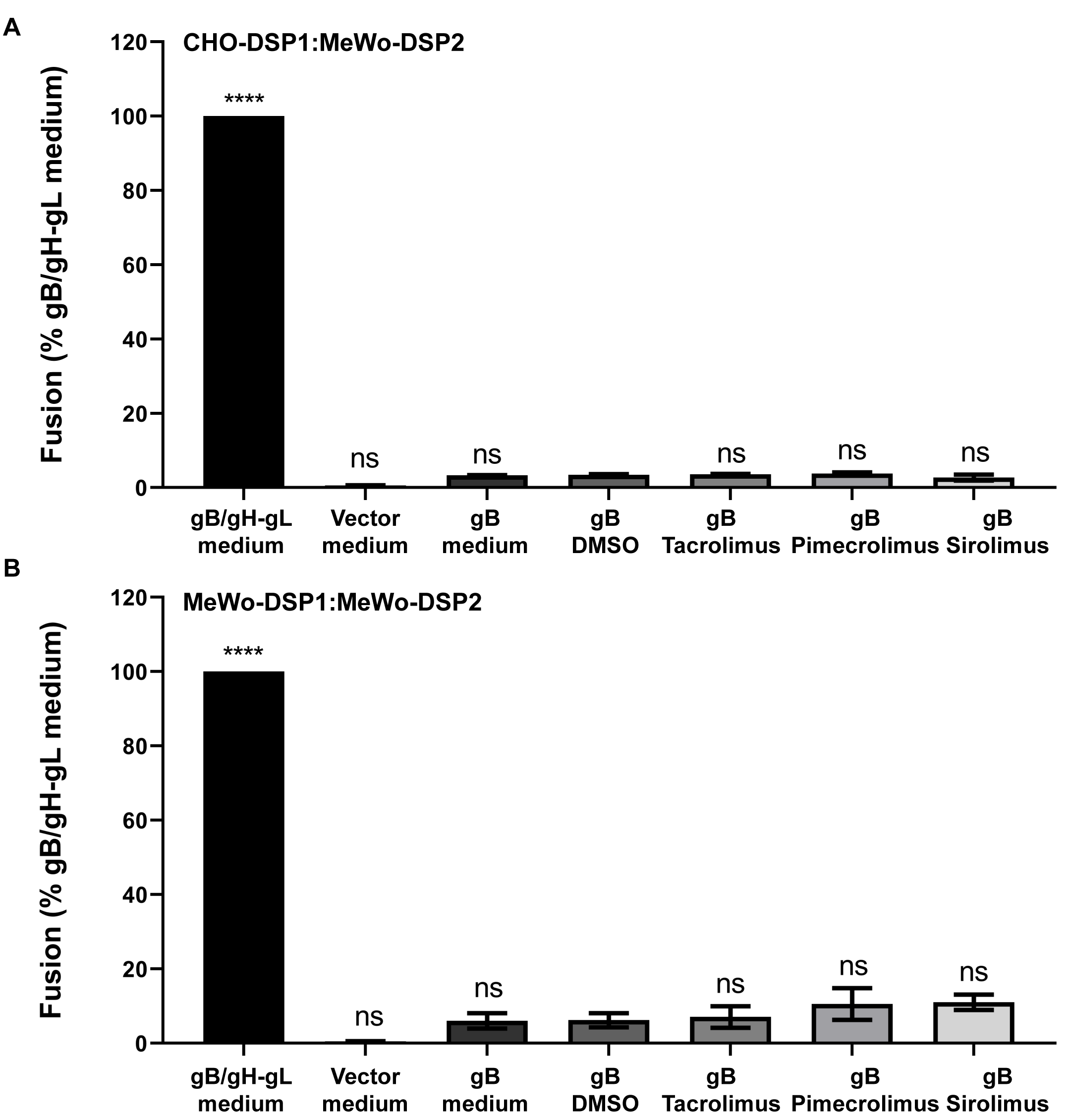

Supplement: S1 Fig — Co-culture of target cells MeWo-DSP2 with effector cells CHO-DSP1 (A) or MeWo-DSP1 (B) transfected with plasmids expressing VZV gB/gH[TL]-gL (gB/gH-gL), empty vectors (Vector) or plasmid expressing gB only (gB) were untreated (medium) or treated with DMSO, or tacrolimus (10 μM), pimecrolimus (10 μM), sirolimus (10 μM) for 48 hrs. Cell fusion efficiency was measured and normalized to that of effector cells transfected with gB/gH[TL]-gL and untreated (% gB/gH-gL medium). Mean ± SEM represent ≥ 3 independent experiments. Statistical differences were assessed by comparison of values to that of effector cells transfected with gB and treated with DMSO using one-way ANOVA (ns, not significant; ****, p < 0.0001). (TIF) [file ppat.1009022.s001.tif]

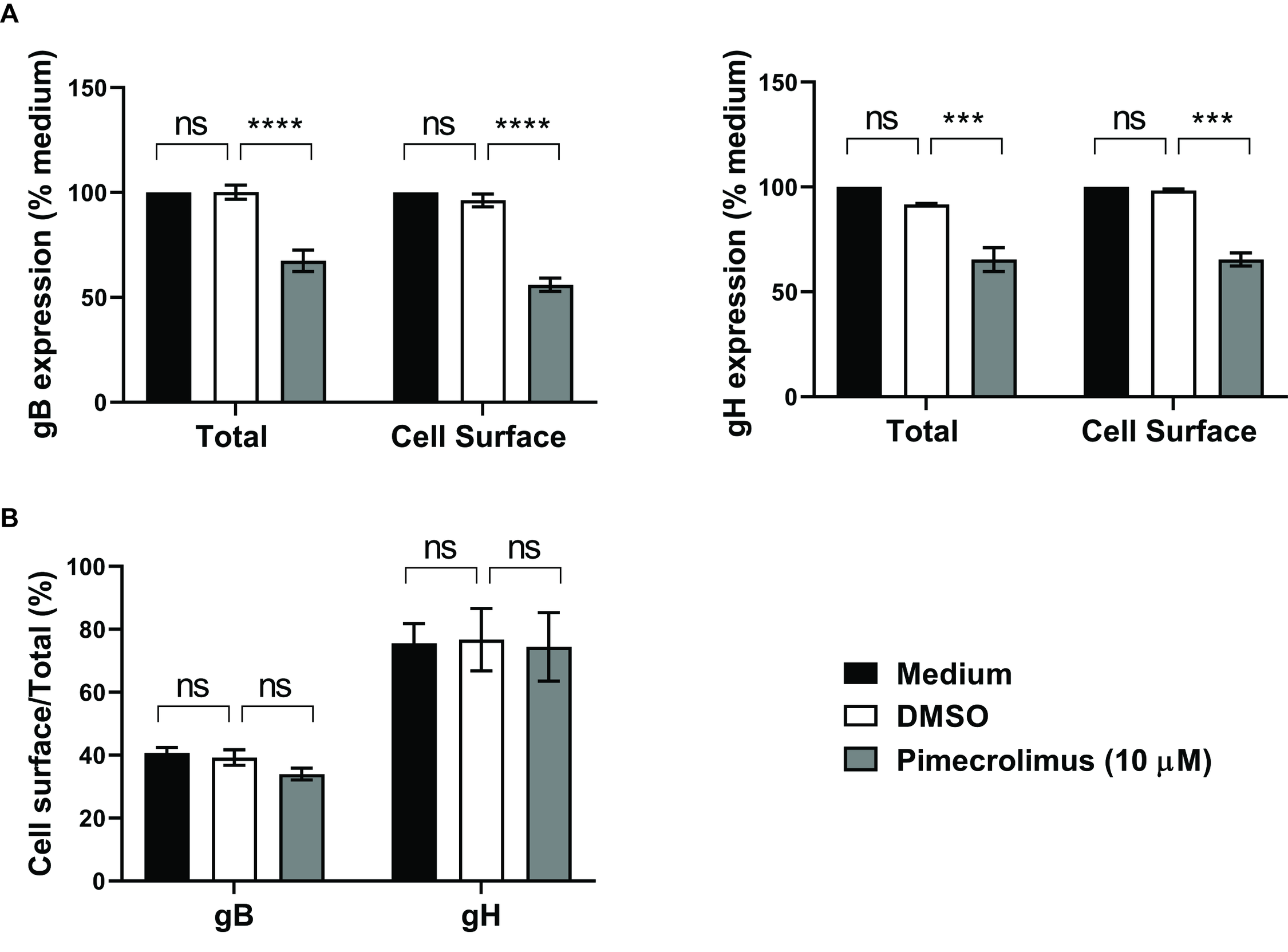

Supplement: S2 Fig — (A) CHO-DSP1 cells transfected with plasmids expressing VZV gB or gH[TL]/gL, were untreated (medium) or treated with DMSO, or pimecrolimus (10 μM) for 24 hrs. The total, and cell surface expression of gB or gH was analyzed by flow cytometry of immunostained permeabilized or nonpermeabilized cells. The population of cells that express gB or gH as total or on the cell surface were normalized to the untreated (% medium) respectively. Mean ± SEM from three independent experiments are shown. (B) The expression level of gB or gH from (A) at cell surface were normalized to the total expression level and presented as a percentage of the total. The brackets represent the statistical differences evaluated by two-way ANOVA (ns, not significant; ***, p < 0.001; ****, p < 0.0001). (TIF) [file ppat.1009022.s002.tif]

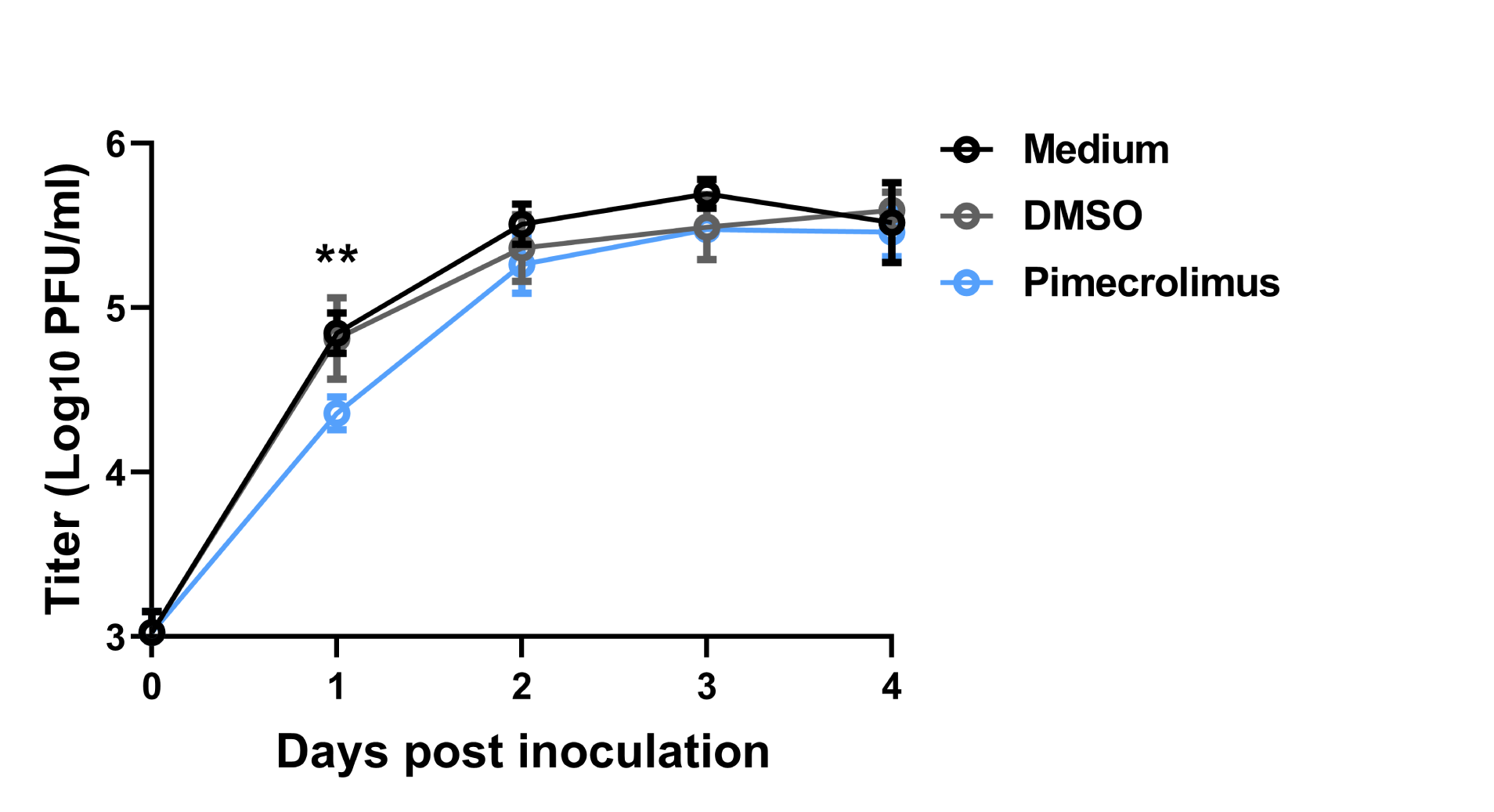

Supplement: S3 Fig — MeWo cells infected with pOka were untreated (medium), treated with DMSO or pimecrolimus (10 μM) for 4 days; media was changed every 48 hrs. Monolayers of infected cells were harvested and titrated on fresh MeWo cells to determine PFU/ml. Representative result from two independent experiments is shown, with mean ± SEM analyzed by two-way ANOVA (**, p < 0.01). (TIF) [file ppat.1009022.s003.tif]
