# Supplementary material for: Calcineurin phosphatase activity regulates Varicella-Zoster Virus induced cell-cell fusion
Source: PLoS Pathog. 2020 Nov 20;16(11):e1009022. doi: 10.1371/journal.ppat.1009022 (PMC7717522; doi:10.1371/journal.ppat.1009022)
Supplement: S3 Table — (DOCX) [file ppat.1009022.s008.docx]

**S3 Table. Calcineurin docking motifs residing on known substrates of calcineurin.**

| **Protein** | **PxIxIT motif** | **LxVP motif** | **Species** | **References** |
| --- | --- | --- | --- | --- |
| NFATC1 | PRIEIT | LAVP | Human | [1] |
| NFATC2 | PRIEIT | LLVP | Mouse | [2] |
| NFATC3 | PSIQIT | LEVP | Human | [3] |
| TORC2 | PNIILT | LVLP | Human | [4] |
| ELK-1 | PEVSVT | LELP | Mouse | [5] |
| MEF2A | PVVSVT | LTVP | Rat | [6] |
| TRESK | PQIVID | LDIP | Mouse | [7] |
| IP3R | PEVKIA | LQIP | Rat | [8] |
| GABA(A) receptor | PAIPVG | IWVP | Rat | [9] |
| K_ATP_ | PSILIQ | LMVP | Human | [10] |
| Kv2.1 | PSLGLY | LPIP | Rat | [11] |
| DARPP-32 | PAMLFR | FSVP | Bovine | [12] |
| KSR2 | PLLQIE | LSVP | Mouse | [13] |
| RACK1 | PSIKIW | YGIP | Human | [14] |
| MAP2 | PDISIT | LMVP | Porcine | [15] |
| Tau | PQVDLE | IPLP | Porcine | [16] |
| Tubulin | PRLHFF | LTVP | Porcine | [16] |
| SSH1 | PALFVD | LVMP | Human | [17] |
| BAD | PNLWAA | FQIP | Human | [18] |
| Drp1 | PQIVVV | LFVP | Human | [19] |

**S3 Table. References**

1. Park S, Uesugi M, Verdine GL. A second calcineurin binding site on the NFAT regulatory domain. Proc Natl Acad Sci U S A. 2000;97:7130-7135.

2. Aramburu J, Garcia-Cózar F, Raghavan A, Okamura H, Rao A, Hogan PG. Selective inhibition of NFAT activation by a peptide spanning the calcineurin targeting site of NFAT. Mol Cell. 1998;1:627-637.

3. Urso K, Fernández A, Velasco P et al. NFATc3 controls tumour growth by regulating proliferation and migration of human astroglioma cells. Sci Rep. 2019;9:9361.

4. Screaton RA, Conkright MD, Katoh Y et al. The CREB coactivator TORC2 functions as a calcium- and cAMP-sensitive coincidence detector. Cell. 2004;119:61-74.

5. Sugimoto T, Stewart S, Guan KL. The calcium/calmodulin-dependent protein phosphatase calcineurin is the major Elk-1 phosphatase. J Biol Chem. 1997;272:29415-29418.

6. Shalizi A, Gaudillière B, Yuan Z et al. A calcium-regulated MEF2 sumoylation switch controls postsynaptic differentiation. Science. 2006;311:1012-1017.

7. Czirják G, Enyedi P. Targeting of calcineurin to an NFAT-like docking site is required for the calcium-dependent activation of the background K+ channel, TRESK. J Biol Chem. 2006;281:14677-14682.

8. Cameron AM, Steiner JP, Roskams AJ, Ali SM, Ronnett GV, Snyder SH. Calcineurin associated with the inositol 1,4,5-trisphosphate receptor-FKBP12 complex modulates Ca2+ flux. Cell. 1995;83:463-472.

9. Wang J, Liu S, Haditsch U et al. Interaction of calcineurin and type-A GABA receptor gamma 2 subunits produces long-term depression at CA1 inhibitory synapses. J Neurosci. 2003;23:826-836.

10. Orie NN, Thomas AM, Perrino BA, Tinker A, Clapp LH. Ca2+/calcineurin regulation of cloned vascular K ATP channels: crosstalk with the protein kinase A pathway. Br J Pharmacol. 2009;157:554-564.

11. Misonou H, Mohapatra DP, Park EW et al. Regulation of ion channel localization and phosphorylation by neuronal activity. Nat Neurosci. 2004;7:711-718.

12. King MM, Huang CY, Chock PB et al. Mammalian brain phosphoproteins as substrates for calcineurin. J Biol Chem. 1984;259:8080-8083.

13. Dougherty MK, Ritt DA, Zhou M et al. KSR2 is a calcineurin substrate that promotes ERK cascade activation in response to calcium signals. Mol Cell. 2009;34:652-662.

14. Liu YV, Hubbi ME, Pan F et al. Calcineurin promotes hypoxia-inducible factor 1alpha expression by dephosphorylating RACK1 and blocking RACK1 dimerization. J Biol Chem. 2007;282:37064-37073.

15. Yamamoto H, Fukunaga K, Goto S, Tanaka E, Miyamoto E. Ca2+, calmodulin-dependent regulation of microtubule formation via phosphorylation of microtubule-associated protein 2, tau factor, and tubulin, and comparison with the cyclic AMP-dependent phosphorylation. J Neurochem. 1985;44:759-768.

16. Goto S, Yamamoto H, Fukunaga K, Iwasa T, Matsukado Y, Miyamoto E. Dephosphorylation of microtubule-associated protein 2, tau factor, and tubulin by calcineurin. J Neurochem. 1985;45:276-283.

17. Wang Y, Shibasaki F, Mizuno K. Calcium signal-induced cofilin dephosphorylation is mediated by Slingshot via calcineurin. J Biol Chem. 2005;280:12683-12689.

18. Wang HG, Pathan N, Ethell IM et al. Ca2+-induced apoptosis through calcineurin dephosphorylation of BAD. Science. 1999;284:339-343.

19. Cereghetti GM, Stangherlin A, Martins de Brito O et al. Dephosphorylation by calcineurin regulates translocation of Drp1 to mitochondria. Proc Natl Acad Sci U S A. 2008;105:15803-15808.
