# Supplementary material for: Calcineurin phosphatase activity regulates Varicella-Zoster Virus induced cell-cell fusion
Source: PLoS Pathog. 2020 Nov 20;16(11):e1009022. doi: 10.1371/journal.ppat.1009022 (PMC7717522; doi:10.1371/journal.ppat.1009022)
Supplement: S4 Table — (DOCX) [file ppat.1009022.s009.docx]

**S4 Table. Primers for shRNA expressing cell construction and RT-qPCR analysis.**

| **Primer Name** | **Sequence (5’ −> 3’)** |
| --- | --- |
| FKBP1A_shRNA_01_Forward | TGCTGTTGACAGTGAGCGCGCCAAACTGACTATATCTCCATAGTGAAGCCACAGATGTA |
| FKBP1A_shRNA_01_Reverse | TCCGAGGCAGTAGGCAAGCCAAACTGACTATATCTCCATACATCTGTGGCTTCACTA |
| FKBP1A_shRNA_02_Forward | TGCTGTTGACAGTGAGCGCAGAGAGCCAAACTGACTATATTAGTGAAGCCACAGATGTA |
| FKBP1A_shRNA_02_Reverse | TCCGAGGCAGTAGGCAAAGAGAGCCAAACTGACTATATTACATCTGTGGCTTCACTA |
| PPP3R1_shRNA_01_Forward | TGCTGTTGACAGTGAGCGCGAGATTTAAGAAGCTTGATTTTAGTGAAGCCACAGATGTA |
| PPP3R1_shRNA_01_Reverse | TCCGAGGCAGTAGGCAAGAGATTTAAGAAGCTTGATTTTACATCTGTGGCTTCACTA |
| PPP3R1_shRNA_02_Forward | TGCTGTTGACAGTGAGCGCGATACACAGTTACAGCAAATTTAGTGAAGCCACAGATGTA |
| PPP3R1_shRNA_02_Reverse | TCCGAGGCAGTAGGCAAGATACACAGTTACAGCAAATTTACATCTGTGGCTTCACTA |
| Adapter-miR30PCRXhoI_Forward | CAGAAGGCTCGAGAAGGTATATTGCTGTTGACAGTGAGCG |
| Adapter-miR30PCREcoRI_Reverse | CTAAAGTAGCCCCTTGAATTCCGAGGCAGTAGGCA |
| Linker-miR30linkerNotI_Forward | ATAAGAATGCGGCCGCGATCCTGCTGTTGACAGTGAGCG |
| Linker-miR30linkerNotI_Reverse | ATAGTTTAGCGGCCGCATCGTAGTCCGAGGCAGTAGGCA |
| pGIPZ-shRNA-sequencing | TTCACCGTCACCGCCGACGTCG |
| FKBP1A-RT-qPCR_Forward | TAAGTTTATGCTAGGCAAGCAGGAG |
| FKBP1A-RT-qPCR_Reverse | ATCCCTCCATGGCAGATCCAA |
| PPP3R1-RT-qPCR_Forward | CCGAGCAAAATGGGAAATGAGG |
| PPP3R1-RT-qPCR_Reverse | AACTCAGGCAGAGACATGAAC |
| PGM1-RT-qPCR _Forward | CCAGAGTATCATCTCCACCGT |
| PGM1-RT-qPCR _Reverse | TGTCCGATAACCAAGCGACC |
