# Supplementary material for: Calcineurin phosphatase activity regulates Varicella-Zoster Virus induced cell-cell fusion
Source: PLoS Pathog. 2020 Nov 20;16(11):e1009022. doi: 10.1371/journal.ppat.1009022 (PMC7717522; doi:10.1371/journal.ppat.1009022)
Supplement: Attachment — Submitted filename: Reply to reviewers.docx [file ppat.1009022.s010.docx]

**Response to the Reviewer’s Comments**

*Author Reply: We thank the Reviewers for their insightful reviews and constructive discussion. Please note, the line numbers and the figure numbers we refer to in this reply are based on the revised clean copy of the manuscript.*

**Part I - Summary**
**Please use this section to discuss strengths/weaknesses of study, novelty/significance, general execution and scholarship.**

Reviewer #1: The manuscript by Zhou et al. screens a panel of molecules to learn more about host cell factors that regulate virus spread, which is a hallmark of VZV pathogenesis. The manuscript is well-written and easy to follow. The data are in general of high quality. Cellular molecules that influence VZV spread are an important topic of investigation. In general, the results are new and interesting but the study seems incomplete, and there are problems with data interpretation and conclusions.

*Author Reply: We appreciate these positive comments and we have replied to the concerns as they are spelled out by this Reviewer in Part II, including the addition of new experiments in this revision.*

Reviewer #2: The experiments described in this manuscript show that calcineurin-phosphatase activity plays a role in regulating VZV-induced cell fusion. This host factor was implicated by the authors' chemical-library screen using cells transfected with the core fusion machinery of the virus. In this experiment, a drug that binds host factor FKBP1A was found to increase cell fusion. That host factor was already known to regulate calcineurin, and the authors nicely show that binding of drug-bound FKBP1A, which blocks phosphatase activity, is needed to increase cell fusion. Thus, some other cellular or viral protein must be de-phosphorylated by calcineurin to keep fusion from getting out of control. The authors also showed that the FKBP1A-binding drug enhanced cell fusion of VZV-infected cells. A variety of other positive and negative inhibitors of fusion were also identified in the screen. Overall, this manuscript makes an important contribution to the literature. Specific comments and concerns are listed below.

1. The experiments that used transfected cells are described in an inaccurate and misleading way. As the authors say in the Methods section, they used a mutant form of gH, which is more fusogenic, but throughout the rest of the text they state that they used "gH", implying that the wild-type was used. To fix this important issue, two changes are needed. First, the rationale for using mutant gH should be stated in the Results section rather than being buried in the Methods. Second, every instance where "gH" is stated in regard to transfections needs to be changed to indicate that the mutant form was used.

*Author Reply: We appreciate this point and have made the following changes to clarify:*

1) *We have described the background from the literature about the more fusogenic nature of gH[TL] in the Introduction, line 107-115. This sets out the rationale for using the mutant gH (denoted as gH[TL]) in our HT-SRFA, which has also been clarified in the Results, line 145-147.*
2) *The use of gH[TL] instead of wild type gH (denoted as gH) in the HT-SRFA and the subsequent experiments done with our standard SRFA assays is described in the Materials and Methods, line 498-502, 550-554, 561-565, and 574-576, and also is indicated in the figure legends wherever it is applicable.*
3) *We would prefer to make the use of gH[TL] under fusion assay conditions clear in the text where we describe how the assay was performed and to keep the simplified designation of the gB/gH-gL fusion assay when we refer to the use of this method. This seems consistent with the Reviewer’s point in comment #3 below, that wild type VZV (pOka)-induced cell fusion was also enhanced by the inhibition of calcineurin phosphatase activity, which confirms that similar enhancing effect due to calcineurin inhibition was also observed in fusion mediated by gB/gH-gL during infection, not just with gB/gH[TL]-gL in the surrogate fusion assay. A sentence related to this rationale was added in Results, line 312-316.*

2. I could not help but wonder why the authors did not examine any of the inhibitory compounds found in their screens. This is particularly "annoying" being that they argue that enhanced fusion reduces virus spread. Would a drug that decreases cell fusion actually enhance cell spread? I find this omission to be a weakness of the study. If there is a convincing reason for it, that should be included in the manuscript.

*Author Reply: Thank you for raising this important point. We have added information to the Introduction, line 103-123 and Discussion, line 389-402 for clarification.*

*Our previous studies have shown that a base level of cell fusion is required for VZV infection, as expected. Mutations of the gB or gH ectodomain that disrupted fusion can cause severe restrictions or inactivate VZV, allowing little or no virus spread (1-3). Thus, we would expect compounds that inhibit fusion would have similar effects on VZV propagation as we observed in evaluating the gB and gH ectodomain mutations. While not included in this report, we have tested a few fusion inhibitors, e.g. tyrphostin AG698, and as expected, they suppressed virus spread. We have included the screening data for these inhibitory compounds since their mechanisms of antiviral activity may be of interest for further study.*

*We chose to focus on host factors that contribute to the regulation of VZV fusion, which is in keeping with our prior studies of gB and gH CTD mediated regulation of this essential process. As we have highlighted in the revision, it is somewhat counterintuitive, but mutations of the gB or gH cytoplasmic domain that interfere with fusion regulation and cause enhanced fusion actually suppressed virus spread outward from plaques in MeWo cell monolayers by their limiting effects on virus particle assembly. Importantly, exaggerated fusion also impaired virus spread in skin xenografts (3, 4). This effect is further illustrated by the introduction of two hyperfusogenic mutations, gB[Y881F] and gH[TL], into the VZV genome, which induced cell-cell fusion but failed to assemble any viral capsids in the nucleus and did not yield infectious VZV (Yang et al., Fig 11, top four panels) (4).*

3. With regard to the virus experiments (and relating to comment #1), it is important to point out that wild-type virus was used, and hence, the drug-induced enhancement of fusion is not dependent of the mutant form of gH. This is important because once readers realize that a gH mutant was used in the transfection/screening experiment, they will of course wonder whether fusion enhancement occurs with wild-type gH. The authors did not address that in the transfections, but the virus experiments perhaps satisfy the question.

*Author Reply: Thanks for this suggestion. We have added a sentence in the Results, line 312-316 to highlight the point that the regulation of calcineurin in VZV gB/gH-gL mediated fusion was observed with WT gH and did not depend on having gH[TL] in the virus.*

4. By the way, reference #30 is incomplete (but I was able to find and read the paper based on its title).

*Author Reply: Thank you for catching this error. The error has been fixed.*

5. Also with regard to the virus experiments, it would be useful to the naive reader to be told (in the paragraph starting on line #235) that cells within syncytia die to create plaques after further incubation (4 days).

*Author Reply: We agree that this may be confusing and have revised the text to clarify. The detachment of the cells in the center of syncytia can be observed as early as 40 hpi and the majority of syncytia had an empty center at 4 dpi, indicating cell lift-off. We have revised the Results, line 327-334, to address this point.*

6. The sentence on lines 246-249 is either misleading or data is missing. The authors state that the higher plaque frequencies observed in drug-treated cultures was "attributed" to detachment of cell membrane fragments and reinfection at secondary locations. What is the evidence for that claim? Did the authors include neutralizing antibodies in the medium to show that small satellite plaques were eliminated. If nothing was done along these lines, then the word "attributed" needs to be replaced with "hypothesize".

*Author Reply: Thank you for bringing this up. We agree that the word “hypothesize” is the correct terminology here, and we have changed the Results section line 327-334. We would note that unlike other neurotropic alphaherpesviruses, for example HSV-1 and PRV, VZV is highly cell-associated so that infectious virus progeny are not released into the supernatant. In our clarification, we propose that fragments of infected cell membranes are produced with the rapid detachment of cells due to exaggerated syncytia development and these fragments can initiate a new round of infection. We also observe formation of microplaques under the typical conditions of VZV infection of MeWo cells but these are much less frequent compared to pimecrolimus treatment.*

7. It is not intuitive to a naive reader why enhanced fusion would result in reduced speading or virus production. They might think that enhanced fusion would enable faster spreading and greater opportunity to make more virus. It would be useful to provide a mechanistic explanation or hypothesis in the Discussion. This is not a new observation but it will be confusing to naive readers.

*Author Reply: We agree that “enhanced fusion results in reduced spread” is not intuitive. Please see our response to the earlier comment #2. We hope this has been clarified for the naïve reader by the changes made in the Introduction, line 103-123, and in the Discussion line 389-402.*

8. The final section of the Results describes mass spectrometry experiments that identified novel substrates for calcineurin. As mentioned above, some protein somewhere in the cell needs to be dephosphorylated to prevent fusion from getting out of control. Here, the authors treated cells with inhibitor and looked for novel proteins that remained phosphorylated. They found five; however, this part of the manuscript is unsatisfying for a few reasons.

*Author Reply: We appreciate this general point and have revised the text to place these findings more clearly in the context of future directions, as well as shortening the Discussion related to these findings.*

8a. The relevance of the five proteins is unclear because only uninfected cells were used. It is abundantly (and nauseatingly) clear that herpesviruses massively alter the physiology of their host cells. Are the five proteins still phosphorylated in drug-treated *infected* cells? Is their expression changed in infected cells? How do we know that these proteins are relevant rather than red herrings?

*Author Reply:*

1) *A barrier to the immediate evaluation of the role of these potential calcineurin substrates in the host cell regulation of VZV fusion was that they are not well-studied to date and antibodies are not commercially available (Table 1 below). However, we have found that mRNA expression levels corresponding to the five proteins as well as the two known substrates NFATC1 and ELK1 that were detected by mass spectroscopy did not differ between VZV infected and uninfected MeWo cells. The mRNA quantities (RPKM values) are provided in Table 2 below and were extracted from the RNAseq data reported in Oliver et al. (5). This strongly suggests that protein levels in infected cells are comparable to those in uninfected cells, but further experiments will be needed to document these or other downstream events that are involved in calcineurin-mediated VZV fusion regulation.*

| **Table 1. Phosphorylation site of the seven uniquely phosphorylated proteins and anti-phospho antibody availabilities.** | | |
| --- | --- | --- |
| Protein | Phosphorylation site | Anti-phospho antibody |
| NFATC1 | S359 | No |
| ELK1 | S324 | No |
| PHACTR2 | S72, S510 | No |
| ILF3 | S482, T486, T504, S477, S503, T368, S382, S384 | No |
| NCOA1 | S369, S698 | No |
| TMC8 | S298, S522 | No |
| MBD1 | S297, S311, S332, S335, S343, T449, T514, S295, T454, | No |

| **Table 2. Expression of genes encoding for the newly identified calcineurin substrates as determined by RNA-Seq in uninfected and VZV infected MeWo cells.** | | | | |
| --- | --- | --- | --- | --- |
| mRNA | Uninfected (RPKM) | Infected  (RPKM) | Log2 (fold_change) uninfected / infected | Statistically significant  difference |
| NFATC1 | 5.989 | 6.540 | 0.127 | no |
| ELK1 | 23.294 | 24.177 | 0.054 | no |
| PHACTR2 | 1.684 | 1.578 | -0.094 | no |
| ILF3 | 75.554 | 71.425 | -0.081 | no |
| NCOA1 | 4.367 | 4.382 | 0.005 | no |
| TMC8 | 0.588 | 0.580 | -0.019 | no |
| MBD1 | 17.569 | 19.026 | 0.115 | no |

*Author Reply:*

*2) In response to the broader concern of the Reviewers that we had not assessed calcineurin phosphatase activity in VZV infected MeWo cells, we have added new data to show that the functional protein is present, as demonstrated using the inonomycin/NFAT nuclear translocation assay (Fig. 5; Results line 293-302). The detection of mRNA transcripts for the potential substrates suggests that they are likely present in infected cells. Importantly, Fig 5 demonstrated that pimecrolimus also inhibited phosphatase activity of calcineurin in VZV infected MeWo cells. Thus it is plausible that pimecrolimus treatment would likely result in persistent phosphorylation of these proteins in VZV infected cells due to inhibition of calcineurin phosphatase activity.*

8b. The authors inappropriately claim on line 276 that they "validated" the five proteins, but they did not. They merely provided further computer-based sequence analyses. To validate their relevance to VZV, they need to actually do experiments!

*Author Reply: We agree that ‘“validated” needs to be softened. Accordingly, we changed the Results, line 354-374, to make it clear that the motif sequence analysis is a speculation on the possibilities of these proteins to be substrates of calcineurin.*

8c. In the context of an infection, there could be critical *viral* targets for calcineurin. This seems to be ignored by the authors, probably because fusion was enhanced in the gB,gH-gL transfection experiments, but viral infections are vastly more complicated. It would not be surprising to find a viral target. This should be addressed.

*Author Reply: As described, our data have demonstrated that calcineurin is pivotal in regulating gB/gH-gL mediated cell fusion when other viral proteins are absent. We have now added new experiments to show that calcineurin inhibition also enhanced cell fusion mediated by the HSV-1 gB//gH-gL/gD core fusion machinery and a host cell fusogen, syncytin-1 (Fig. 2G; Results section line 238-253). Thus, we conclude from this generalized effect that calcineurin is likely to regulate cell fusion through differential phosphorylation of host cell factors, regardless of whether cell fusion is mediated by a viral fusion complex or a host cell fusogen. It is possible that a viral glycoprotein target could also contribute but the host cell network appears to be sufficient for calcineurin dependent regulation.*

8d. A large portion of the Discussion is devoted to these five proteins, but it is unsatisfying because the experiments are incomplete and premature. Would other investigators benefit from the data in its current form? Yes. I do not think it should be eliminated from the manuscript. But it does not deserve so much attention, and the limitations need to be pointed out. All the detailed Discussion can be removed. Any investigator who wishes to pursue these five proteins will have no problem finding the relevant background information.

*Author Reply: We have shortened the Discussion on the potential substrates of calcineurin as recommended. However, Reviewer #3 found the analysis of the newly identified proteins that may be targeted by calcineurin to be useful and we agree that others might find it useful as background for future studies in addition to examining the databases themselves. To address the Reviewer’s concern, we have moved some details to the S2 Table instead of deleting the information altogether.*

9. Also in the Discussion, the authors mention previous experiments with HSV-1 showing that a different phosphatase (PTP1B) can regulate cell fusion. My reading of that paper reveals the need for a couple of clarifications here.

9a. Unlike VZV, the HSV-1 fusion machinery in isolation (via transfection) is highly efficient and does not require the use of mutant proteins. Nevertheless, it might be interesting to ask whether inhibition of calcineurin activity would have an effect on HSV-1, and that idea could be added to the Discussion.

*Author Reply: Thanks for asking this question, which prompted us to undertake new experiments about the effect of calcineurin inhibition on HSV-1 glycoproteins-mediated cell fusion. This new data is mentioned in response to comment #8c above. Indeed, calcineurin was found to regulate fusion driven by HSV-1 glycoproteins as well as VZV (Fig. 2G; Results section line 238-253).*

9b. Wild-type HSV-1 is not fusogenic. Inhibitors of PTP1B did not enhance fusion. Rather, they prevented the virus from spreading to adjacent cells. I think the HSV-1 study used the artificial situation of cell fusion to get at the mechanism of cell-to-cell spread. Would inhibition of calcineurin activity after HSV-1 spread? That idea could be mentioned in the Discussion.

*Author Reply: We agree that wild type HSV-1 is much less fusogenic than VZV in cultured cells, although multi-nucleated cells are observed clinically in some HSV-1 mucocutaneous lesions. HSV-1 syncytium-forming variants can arise in cell culture and have been noted with gH mutations (6-9). We did not test whether calcineurin phosphatase regulates HSV-1 fusion during infection, but we would predict that it would from our observations with the glycoprotein-based fusion assay, now added to our Results, Fig 2G, line 238-253.*

9c. While the paper showed that HSV-1 is induced to fuse cells when treated with salubrinal (a phenotype that was blocked by inhibitors of PTP1B), the authors showed that the target of salubrinal is *not* eIF2a. Thus, it appears that the target remains unknown.

*Author Reply: Thank you for catching this error. Upon re-reading this paper, our understanding on this point is that salubrinal blocks the dephosphorylation step of eIF2α which has been phosphorylated during ER stress, thus inhibits protein translation and prolongs the cell response to ER stress, but the target of the drug is unknown. Therefore, the authors used the phosphorylation of eIF2α as a read-out to test whether PTP1B is salubrinal’s direct target. They found that phosphorylation of eIF2α in response to salubrinal still occurred in the absence of PTP1B, indicating that PTP1B is not the target of salubrinal. Accordingly, the text was revised in the Discussion, line 456-459.*

10. With regard to the Supporting Information, I think S3-Fig is of critical importance and should be included with the main set of figures. Of course, this brings to greater attention the hand-waving on lines 249-250 regarding the "apparent equivalence of VZV growth kinetics", which is also mentioned in comment #6, above.

*Author Reply: We appreciate this recommendation and we have moved Fig. S3 to the main figures as Fig 7D. We also revised the text in the Results, line 325-336. We also hope that we have addressed this concern in our revisions as described in our response to comment #6,*

Reviewer #3: With this manuscript, Arvin, Oliver and colleagues report that inhibition of calcineurin’s phosphatase activity leads to enhanced VZV glycoprotein-mediated cell-cell fusion. They also identify seven host proteins that are phosphorylated at serine or threonine sites if calcineurin’s phosphatase activity is inhibited, suggesting that they might be contributing to regulation of VZV-induced cell-cell fusion, thus securing efficient virus spread.
Overall the data are of good quality, well presented, and appropriately discussed. While the paper does not yet report on studies that start testing if any of those newly identified potential downstream targets of calcineurin’s phosphatase activity is involved in regulating VZV-induced cell-cell fusion, this report of currently available data should still be of interest for others studying virus-induced cell-cell fusion processes. In the following, I thus merely list points that might assist in further improving the manuscript.

*Author Reply: We appreciate this positive assessment of our work.*

Title, Abstract, Introduction, and Discussion

Title and Abstract appropriately summarize the results presented in this paper. The authors also provide a fine introduction into previous investigations of VZV-induced cell fusion and an appropriate, if somewhat short, discussion of the data presented in this report. While this reviewer typically prefers if authors focus most of the discussion on the data that are presented, in this case I think it is appropriate to speculate how one or the other of the newly identified downstream targets of calcineurin may be contributing to regulation of VZV-induced cell fusion.
Minor point: given that their HT-SRFA yields many potential cellular targets that could potentially be factors involved in fusion regulation, it would be interesting to learn why the authors chose to hone in on calcineurin. Was it because of calcineurin’s role in regulating cellular cell-cell fusion processes which is described in the Discussion? If so, I think it would be appropriate to mention this briefly already at the end of the Introduction.

*Author Reply: We appreciate the Reviewer’s interest in why we focused on calcineurin. We have revised the text to explain this point in the Results, line 164-182.*

*Briefly, in our original review of the HT-SFRA data, we noted that there were several calcium channel inhibitor drugs that enhanced VZV gB/gH-gL mediated cell fusion. Since cytosolic Ca^2+^ concentration is important for various cellular signaling pathways and regulating the activity of many enzymes, we looked for other hits from the HT-SRFA screening that targeted a more specific host protein(s) responsive to changes in Ca^2+^ concentration while having an analogous effect on VZV gB/gH-gL fusion as the calcium channel inhibitors did. We identified tacrolimus (FK506) as a fusion enhancer, which is well-known as inhibitor of calcineurin. Tacrolimus acts by binding to the host protein FKBP1A and the drug-protein complex then binds to calcineurin, specifically blocking its phosphatase activity. Calcineurin, a serine/threonine phosphatase, is activated by Ca^2+^. Thus, the inhibitory effect of tacrolimus/FKBP1A binding on calcineurin parallels that of decreased cytosolic Ca^2+^ concentration by calcium channel inhibitor drugs with regard to fusion enhancement.*

*We had not appreciated what was known about calcineurin in host cell fusion before analyzing the VZV HT-SFRA data. As we describe in the Discussion line 403-418, while calcineurin has not been reported to regulate virus-induced cell fusion, the enzyme has been found to play a role in regulating host cell-cell fusion under the few circumstances when host cells undergo cell-cell fusion. As noted, the new experiments we have added to this revised manuscript extend our original finding by showing that the effect of the inhibition of calcineurin function is generalized for the HSV-1 fusion complex as well as syncytin-1, which is derived from an endogenous retrovirus and involved in placental trophoblast formation (Fig 2G, Result, line 238-253).*

Results

The results section is overall well written, clear, and organized, findings are not overstated.

Minor points, suggestions, comments:

The authors address grouping of fusion enhancers that have a link to neurotransmission. It might be interesting to try their fusion assays using neural cells? (obviously beyond the scope of this paper, but this might provide intriguing additional information, given that previous work of one of the senior authors, PMID 18256143, documented VZV-induced fusion of neurons and satellite cells)

*Author Reply: We agree that this will be an intriguing question for further study. At present, we have not developed an in vitro system that could be used for these experiments.*

Lines 173-174: when discussing glycoprotein expression levels (total and surface), the authors write that they were “lower in transfected CHO-DSP1 cells treated with pimecrolimus compared to medium or DMSO treatment (S2A Fig),”. It might be useful in this case to have a control stain to show why this might be. Are the cells more resistant to transfection, or does pimecrolimus elicit a larger, non-specific disruption to normal protein production? This would be useful to know as they continue to use the pimecrolimus treatment down the line in the plaque assays and titration curves.

*Author Reply: We address the questions as follows:*

1) *“Are the cells more resistant to transfection?” For the surface staining experiment, we transfected the CHO cells first and waited 6 hrs before treatment with pimecrolimus. Given the experimental procedure and the timing for adding the drug, it is unlikely that cells are more resistant to transfection because by the time they are treated, plasmids should already have entered the cells and started to express proteins. For example, by 6 hrs, cells transfected by NFATC1-GFP plasmid already expressed the GFP signal.*
2) *“Does pimecrolimus elicit a larger, non-specific disruption to normal protein production?” If a severe defect in protein production was caused by pimecrolimus some level of toxicity or cell growth defect would have been detected. We did not find evidence of a generalized effect on protein production based on careful monitoring for drug toxicity (Fig. 2D). In addition, cell growth defects were not evident in our plaque assays, which are performed over 4 days.*

The authors nicely differentiate between the various potential mechanisms of FKBP1A (impacting calcineurin vs impacting mTOR using specific inhibitors with known function), and validate the impact on calcineurin by multiple approaches.

*Author Reply: Thanks!*

Line 204: the authors mention a “random shRNA control”, but unless I miss it, there is no description of that sequence in the methods, nor do they describe the generation of such a construct?

*Author Reply: Thank you for noting this oversight. The shRNA control is a pGIPZ Non-Silencing Control Vector that is included in the Thermo Scientific Open Biosystems TransLenti Viral GIPZ Packaging System. This was used as expression control to generate non-silencing lentiviral stock to optimize expression conditions in the MeWo cells for the shRNA constructs. We have changed our text in the Materials and Methods in line 590-619 to better describe the experimental procedure, and also changed the phrase “random shRNA control” to “control cells” in the main text and the figure legend.*

Lines 218-221: “These findings corroborated the NFATC1 nuclear translocation data, indicating that FKBP1A was the specific cellular protein bound by pimecrolimus to produce the inhibitory effect on calcineurin and confirmed that dysregulation of VZV gB/gH-gL mediated cell fusion by pimecrolimus was a result from the inhibition of calcineurin phosphatase activity”. I can see how the authors are connecting these dots, but I think they should complete experiments to at least investigate some metric of calcineurin activity within the context of an actual VZV infection rather than in uninfected cells, or they should soften their statement here (specifically as they use the phrasing “confirmed that dysregulation of VZV”).

*Author Reply: We appreciate this important point and we have now tested calcineurin phosphatase activity in VZV infected MeWo cells using the NFATC1 nuclear translocation assay. We found that NFATC1 was distributed in the cytoplasm of VZV infected cells without drug and that ionomycin treatment induced NFATC1 nuclear translocation whereas pimecrolimus treatment prevented NFATC1 translocation. The new data was added as Fig.5 and text was added in the Results, line 293-302.*

Figure 3B labelling suggests that nuclei were labeled with DAPI, yet the legend says Hoechst. A quick search shows no mention of DAPI in the text, assuming this was a typo?

*Author Reply: Thanks – this was a typo which has been corrected.*

Figure 3B shows difference between localization within the KD or control, but doesn’t show how baseline localization may vary between KD and control which would be interesting to know. The way the data was normalized makes it impossible to know. Also, since the data in 3B are from two replicates, there should not be error bars and statistical analysis. Rather, the individual replicates should be shown. Same goes for C where there are only 3 replicates.

*Author Reply: Thanks for this suggestion. We have converted Fig 3B to box-whiskers plot to better represent the distribution and median of the individual data. We also modified the figure legend accordingly. We kept the mean ±SEM and significant difference pairs because we think the information will be clearer to the reader in this format. The baseline localization of NFATC1 in control and FKBP1A KD cells was similar as shown in Fig 3B, “untreated” condition.*

The discussion of S3Fig A and B is out of order. Perhaps one could reorganize the figure or the discussion of the results so that the two pieces are more reflective of each other?

*Author Reply: Thanks for catching this mistake. We now moved S3 Fig B to Fig 7D and changed the text in Results section, line 327-336.*

Fig. 4C: please show the individual data points. Ideally, avoid statistical testing, but if needed (i.e. if the result is not abundantly clear in the dot plot), it should at least be nonparametric unless normal distribution can be demonstrated.

*Author Reply: This is a very good suggestion. We changed this figure accordingly to now Fig 6C with individual data points plotted and we have analyzed the statistical difference by nonparametric Mann-Whitney test. The statistical difference was observed with this method as well.*

Fig. 5B: not sure if it is appropriate here to take each plaque as a data point, there really are only two replicates here, hence statistics and error bars may not be appropriate?

*Author Reply: Fig 5B is now Fig 7C and was revised to present the data in box-whiskers plot, showing the distribution, median and mean.*

Materials and Methods
**Lines 397-401** describe that the authors used a CTD-mutant gH protein for their screening protocol (and presumably also for some of the subsequent assays?). However, as mentioned in the Introduction, the CTD of gB and gH affects fusion regulation and even cellular expression profiles. While in subsequent experiments the effects on fusion and plaque formation are shown with WT gH-expressing virus, thus validating them, usage of mutant gH should nevertheless be mentioned in the Results section.

*Author Reply: This important point was noted by Reviewer #2 in comment #1. It has been addressed in the revised text as described in the reply to this Reviewer. Changes have been made in the Introduction, line 107-115 and the Results, line 145-147.*

**Part II – Major Issues: Key Experiments Required for Acceptance**
Please use this section to detail the key new experiments or modifications of existing experiments that should be absolutely required to validate study conclusions.

Generally, there should be no more than 3 such required experiments or major modifications for a "Major Revision" recommendation. If more than 3 experiments are necessary to validate the study conclusions, then you are encouraged to recommend "Reject".

Reviewer #1: 1) If the authors would like to make this study about calcineurin, then its role in VZV fusion should be directly tested, e.g. by knockdown. The conclusions about calcineurin and VZV are overstated because the approaches are quite indirect. The inhibitors in the study, pimecrolimus and related molecules, target FKBP1A, not calcineurin itself. One could also argue that the big picture story here is more about calcium flux and less about calcineurin or FKBP1A.

*Author Reply: We respectfully counter that the comment “pimecrolimus and related molecules, target FKBP1A, not calcineurin itself” is inaccurate. Pimecrolimus and tacrolimus (FK506) are immunosuppressant drugs with well-established mechanisms and have been widely studied in T cells. Both drugs form a complex with FKBP1A, which subsequently binds to and inhibits calcineurin phosphatase activity, thus blocking the dephosphorylation of NFAT required for IL-2 transcription in T cell activation and proliferation. A crystal structure of the tacrolimus/FKBP1A/calcineurin complex reveals the binding of tacrolimus/FKBP1A to calcineurin and that it physically blocks the access of the substrate for calcineurin to its active site (10, 11). More recent studies also demonstrated that the binding site of tacrolimus/FKBP1A on calcineurin overlaps with one of the substrate docking sites on calcineurin (via the LxVP motif), thus providing additional molecular explanation of how these drugs interfere with calcineurin’s activity by blocking LxVP containing substrates from binding to calcineurin (12). Taken together, there is strong biochemistry and structural evidence that both pimecrolimus and tacrolimus target calcineurin specifically by forming a drug-protein complex with FKBP1A.*

*Since it had not been reported previously to our knowledge, we verified this mechanism of action in MeWo cells before proceeding with our experiments to use pimecrolimus and tacrolimus as a probe to evaluate the phosphatase activity of calcineurin in regulating VZV gB/gH-gL mediated cell fusion. Further, we included another drug, sirolimus, which is known to bind to FKBP1A but directs the drug-protein complex to bind to mTOR rather than calcineurin (13, 14). We compared tacrolimus/pimecrolimus versus sirolimus with several approaches, including biochemical interaction by IP-WB and NFATC1 nuclear translocation assay as well as showing no effect of sirolimus on the regulation of gB/gH-gL fusion.*

*As noted above, our HT-SRFA identified several calcium channel blocker drugs as fusion enhancers, which supports the likely contribution of calcium flux. However, we have discovered an unreported role of the host cell factor, calcineurin, in regulating virus-induced cell fusion. Moreover, although calcineurin is a Ca^2+^-responsive enzyme, pimecrolimus and tacrolimus do not interfere with the Ca^2+^ binding to CNB subunit, a necessary step required for calcineurin activation (10, 11).*

*As recommended, we have now tested the effect of calcineurin knockdown (KD) on VZV gB/gH-gL mediated cell fusion. New data is added in Fig. 4 and text has been added in the Results, line 279-292. These findings indicate that the intact calcineurin heterodimer is important for VZV gB/gH-gL to mediate fusion.*

The identification of host cell substrates for calcineurin in MeWo cells is interesting from a cell biology perspective, but there is no evidence that these substrates have anything to do with VZV infection. The paper does not test the role of these substrates in VZV fusion and spread.

*Author Reply: To address this important point, we have now shown the phosphatase activity of calcineurin in VZV infected MeWo cells by NFATC1 nuclear translocation assay. Ionomycin treatment induced NFATC1 nuclear translocation in VZV infected cells which otherwise was distributed in the cytoplasm, and more importantly, pimecrolimus treatment prevented this translocation. New data was added as Fig. 5 and text was added in the Results, line 293-302.*

*We have addressed the more general comment about the mass spectroscopy data in our reply to Reviewer 2’s comment # 8 above. This Reviewer raised similar concerns. We have responded by putting this information in the context of future directions of interest and by reducing the extent of the Discussion accordingly.*

3)The terms fusion and spread as used here are a bit misleading and confusing. A larger plaque size means the plaque encompasses a larger area or volume, but does not mean spread to more cells. The results presented exemplify this. The number of nuclei that comprise a syncytium is a more accurate reflection of both the ability of a transfected/infected cell to fuse with AND spread to other cells in the culture.

*Author Reply: We appreciate the request for clarification on this subject. It is obvious that our explanation was inadequate since the same concern is raised by Reviewer 2, comment #2 and comment #7 above. Please see our response to review 2’s comment #2 above about the need to clarify the complex interplay of cell-cell fusion in the context of VZV infection. Changes were made in the Introduction, line 103-123; Discussion, line 389-402.*

*We agree that the area covered by one plaque could be the same size if individual infected cells in a cluster each expanded or alternatively, if the number of infected cells increased so that there were more infected cells involving an area of equivalent size.*

*We also agree that the number of nuclei that comprise a syncytium is a relevant measure for fusion. However, for VZV, the capacity of the virus to spread is not equivalent to its ability to fuse. VZV infects neighboring cells in the monolayer by being externalized on to cell surfaces in proximity to the plasma membrane of the neighboring cell at early times after infection whereas the normally regulated process of fusion is first noted about 9 hours after infection (15). We hope that we have now explained how exaggerated fusion actually impairs VZV spread because it restricts the assembly of progeny viruses. As noted in response to Reviewer #2, the gB[Y881F]/gH[TL] in Yang et al., 2014 illustrates how the induction of cell-cell fusion by VZV genomic DNA is separate from the capacity to produce infectious virus.*

Inhibition of calcineurin phosphatase activity enhanced VZV-induced cell fusion but reduced VZV spread. These disparate results are not clearly addressed. If there is enhancement in one assay and decrease in another, then the assays are not measuring the same thing. We are left without a sense of what a decrease in syncytium size really means (if it is not related to a decrease in fusion) and why it is relevant to VZV pathogenesis.

*Author Reply: The Reviewer is correct that the assays measure different events. VZV-induced cell fusion was measured by the number of nuclei in syncytium in Fig. 6 while VZV spread was measured by plaque size in Fig. 7. We have tried to clarify this point in the revised text in result section line 303-336.*

Reviewer #2: None

Reviewer #3: n/a

**Part III – Minor Issues: Editorial and Data Presentation Modifications**
Please use this section for editorial suggestions as well as relatively minor modifications of existing data that would enhance clarity.

Reviewer #1: The manuscript assesses chemical inhibitors and knockdown of FKBP1A on VZV cell fusion and plaque size, yet the background on FKBP1A and its role in the cell, relationship to calcineurin, and calcium is buried. There should be more focus on FKBP1A in the writing. It should be better described in the abstract and introduction.

*Author Reply: We have responded to this concern in our revisions to the text and as outlined in our reply to Review 1’s comment #1 in Part II above. Revisions appear in Result, line 169-182 and Discussion, line 377-388.*

Reviewer #2: None beyond what was mentioned in the review

Reviewer #3: included in Part I

***References***

*1.	Oliver SL, Sommer M, Zerboni L, Rajamani J, Grose C, Arvin AM. Mutagenesis of varicella-zoster virus glycoprotein B: putative fusion loop residues are essential for viral replication, and the furin cleavage motif contributes to pathogenesis in skin tissue in vivo. J Virol. 2009;83(15):7495-506.*

*2.	Vleck SE, Oliver SL, Brady JJ, Blau HM, Rajamani J, Sommer MH, et al. Structure-function analysis of varicella-zoster virus glycoprotein H identifies domain-specific roles for fusion and skin tropism. Proc Natl Acad Sci U S A. 2011;108(45):18412-7.*

*3.	Oliver SL, Brady JJ, Sommer MH, Reichelt M, Sung P, Blau HM, et al. An immunoreceptor tyrosine-based inhibition motif in varicella-zoster virus glycoprotein B regulates cell fusion and skin pathogenesis. Proc Natl Acad Sci U S A. 2013;110(5):1911-6.*

*4.	Yang E, Arvin AM, Oliver SL. The cytoplasmic domain of varicella-zoster virus glycoprotein H regulates syncytia formation and skin pathogenesis. PLoS Pathog. 2014;10(5):e1004173.*

*5.	Oliver SL, Yang E, Arvin AM. Dysregulated Glycoprotein B-Mediated Cell-Cell Fusion Disrupts Varicella-Zoster Virus and Host Gene Transcription during Infection. J Virol. 2017;91(1):e01613-16.*

*6.	Little SP, Jofre JT, Courtney RJ, Schaffer PA. A virion-associated glycoprotein essential for infectivity of herpes simplex virus type 1. Virology. 1981;115(1):149-60.*

*7.	Bond VC, Person S, Warner SC. The isolation and characterization of mutants of herpes simplex virus type 1 that induce cell fusion. J Gen Virol. 1982;61 (Pt 2):245-54.*

*8.	Romanelli MG, Cattozzo EM, Faggioli L, Tognon M. Fine mapping and characterization of the Syn 6 locus in the herpes simplex virus type 1 genome. J Gen Virol. 1991;72 ( Pt 8):1991-5.*

*9.	Tognon M, Guandalini R, Romanelli MG, Manservigi R, Trevisani B. Phenotypic and genotypic characterization of locus Syn 5 in herpes simplex virus 1. Virus Res. 1991;18(2-3):135-50.*

*10.	Griffith JP, Kim JL, Kim EE, Sintchak MD, Thomson JA, Fitzgibbon MJ, et al. X-ray structure of calcineurin inhibited by the immunophilin-immunosuppressant FKBP12-FK506 complex. Cell. 1995;82(3):507-22.*

*11.	Kissinger CR, Parge HE, Knighton DR, Lewis CT, Pelletier LA, Tempczyk A, et al. Crystal structures of human calcineurin and the human FKBP12-FK506-calcineurin complex. Nature. 1995;378(6557):641-4.*

*12.	Grigoriu S, Bond R, Cossio P, Chen JA, Ly N, Hummer G, et al. The molecular mechanism of substrate engagement and immunosuppressant inhibition of calcineurin. PLoS Biol. 2013;11(2):e1001492.*

*13.	Dumont FJ, Staruch MJ, Koprak SL, Melino MR, Sigal NH. Distinct mechanisms of suppression of murine T cell activation by the related macrolides FK-506 and rapamycin. J Immunol. 1990;144(1):251-8.*

*14.	Ballou LM, Lin RZ. Rapamycin and mTOR kinase inhibitors. J Chem Biol. 2008;1(1-4):27-36.*

*15.	Reichelt M, Zerboni L, Arvin AM. Mechanisms of varicella-zoster virus neuropathogenesis in human dorsal root ganglia. J Virol. 2008;82(8):3971-83.*
